# A case report on atypically presented Congenital Solitary pelvic kidney with neglected Ureteropelvic junction obstruction (UPJO) successfully managed with open Anderson-Hynes pyeloplasty. Case report

**DOI:** 10.1016/j.ijscr.2025.111603

**Published:** 2025-07-04

**Authors:** Abiy Tadele Alene, Habtamu Aderaw Zeru, Tensae Tesfaye Amare, Biniam Goa Mammo, Yohannes Kifle Tesema, Biniam Yohannes Kassa

**Affiliations:** a Final year urology Resident, Addis Ababa University, College of Health Sciences, School of Medicine, Ethiopia; b Assistant Professor of Urology, Addis Ababa University, College of Health Sciences, School of Medicine, Ethiopia; c Final year pathology resident, Addis Ababa University, College of Health Sciences, School of Medicine, Ethiopia; d Urology resident, Addis Ababa University, College of Health Sciences, School of Medicine, Ethiopia; e Final year internal medicine resident, Addis Ababa University, College of Health Sciences, School of Medicine, Ethiopia

**Keywords:** Case report, Solitary kidney, Pelvic kidney, Ureteropelvic junction obstruction, Anderson-Hynes Pyeloplasty

## Abstract

**Introduction and importance:**

Kidney birth defects rank second to cardiac/skeletal anomalies. Horseshoe kidney is the most common renal fusion anomaly; pancake/lump kidney is the rarest. Congenital anomalies of the kidney and urinary tract (CAKUT), a spectrum varying in severity, include pelvic kidney and UPJO, major contributors to pediatric ESRD.

To our knowledge, congenital solitary pelvic kidney with UPJO has not been reported. Here, we present a 13-year-old with congenital solitary pelvic kidney and neglected UPJO, atypical presentation, successfully managed with open Anderson-Hynes (A-H) pyeloplasty. This report follows updated Surgical CAse REport (SCARE) guidelines [14].

**Clinical presentation:**

A 13-year-old from the Somali region of Ethiopia presented with 7-month history of vague lower abdominal pain, worsening over 2 months, associated with low grade intermittent fever, weight loss, anorexia, uneasiness, and fatigue.

**Clinical discussion:**

Pelvic kidney, a birth defect where kidneys fail to ascend, often presents asymptomatically but increases risk of trauma, UTIs, stones, and urological issues due to location/drainage. UPJO, obstructing urine flow from renal pelvis to ureter (intrinsic: adynamic segments/valves; extrinsic: crossing vessels), causes hydronephrosis/renal damage. Prenatal ultrasound detects UPJO; adults are often diagnosed incidentally. Evaluation involves history, examination, and imaging (US, CT urography, renal scintigraphy) to plan intervention. Symptomatic UPJO with obstruction/declining function warrants pyeloplasty (open/laparoscopic/robotic). Endoscopic approaches (endopyelotomy) may also considered.

Congenital solitary pelvic kidney complicated by UPJO is a rare, challenging scenario with substantial risk of progressive renal parenchymal loss and declining renal function, emphasizing the need for evaluation.

**Conclusion:**

Congenital solitary pelvic kidney is a rare condition that can be complicated by UPJO, potentially leading to loss of renal function. In pelvic kidney with UPJO, imaging may not be straightforward, particularly with late presentation. UPJO should be considered as a cause of renal function loss in patients with pelvic kidney. Early diagnosis and management are crucial for preserving renal parenchyma**.**

## Highlights

• Birth defects affecting the kidneys are frequent, they are leading causes of end-stage renal disease (ESRD) in pediatric patients.
• Pelvic kidney, resulting from failed kidney ascent, increases risk for urological complications, most of the time they are asymptomatic.
• Ureter pelvic junction obstruction (UPJO) frequently causes urinary drainage blockage, which subsequently result in renal injury and damage.
• The combination of a solitary kidney located in the pelvis and UPJO is a rare occurrence that may result in significant renal damage before diagnosis due to their asymptomatic nature.
• Early diagnosis and management of UPJO in solitary pelvic kidneys are crucial to early preserve renal parenchyma. Imaging and presentation may not be typical so, we need to keep in mind the possibility of UPJO in solitary pelvic kidney with hydronephrosis.

## 1 Clinical presentation

### 1.1 History and physical examination

A 13-year-old male adolescent from the Somali region of Ethiopia presented with seven months of left lower vague abdominal pain which was intermittent and mild, but worsened over the past two months in frequency and severity associated with low grade intermittent fever, fatigue, malaise, anorexia, significant weight loss, nausea, and vomiting.

He and family denied urinary symptoms and had no family history of similar illnesses.

He was a fifth-grade student with normal growth prior to the onset of symptoms.

Two weeks prior to presentation to our hospital took antibiotics and a 5fr DJ stent was inserted for obstruction diversion and pyonephrosis. Two weeks of stent insertion, Creatinine trended down to 1.7 mg/dL and he improved clinically.

Upon our evaluation.

On physical examination vital signs blood pressure 100/60mmhg, pulse rate; 92, oxygen saturation; 94 % with atmospheric air.

Abdominal examination revealed a non-tender cystic mass in the left lower quadrant, the borders of which were ill-defined. The remainder of the physical examination was unremarkable.

### 1.2 Investigations

Complete blood count (CBC) revealed: white blood cell count 10,000 cells/dL, hemoglobin (HGB) 12 g/dL, and platelet count 250,000 cells/dL. Urinalysis showed +3 leukocytes with many WBCs, but no RBCs. Urine culture showed no growth after 24 h. Initial serum creatinine was 1.8 mg/dL.

**Fig. 1:**
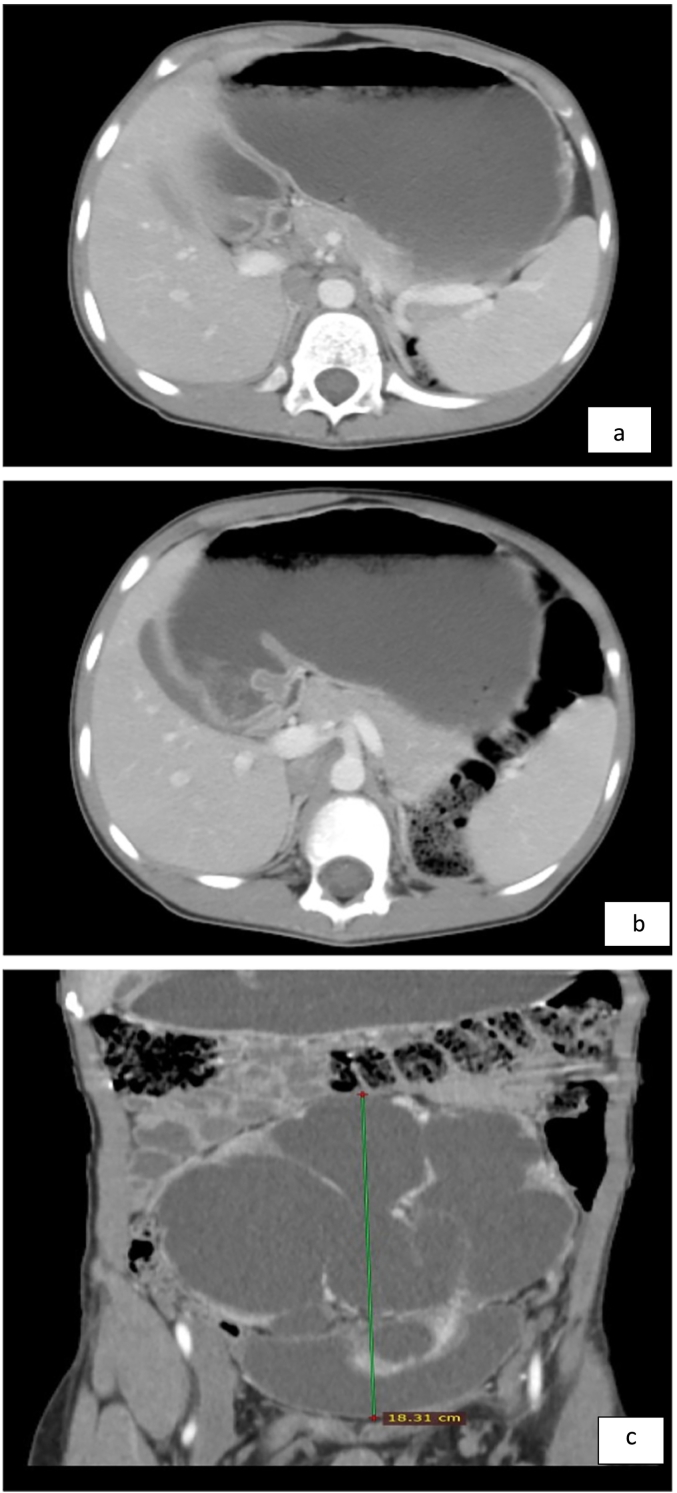

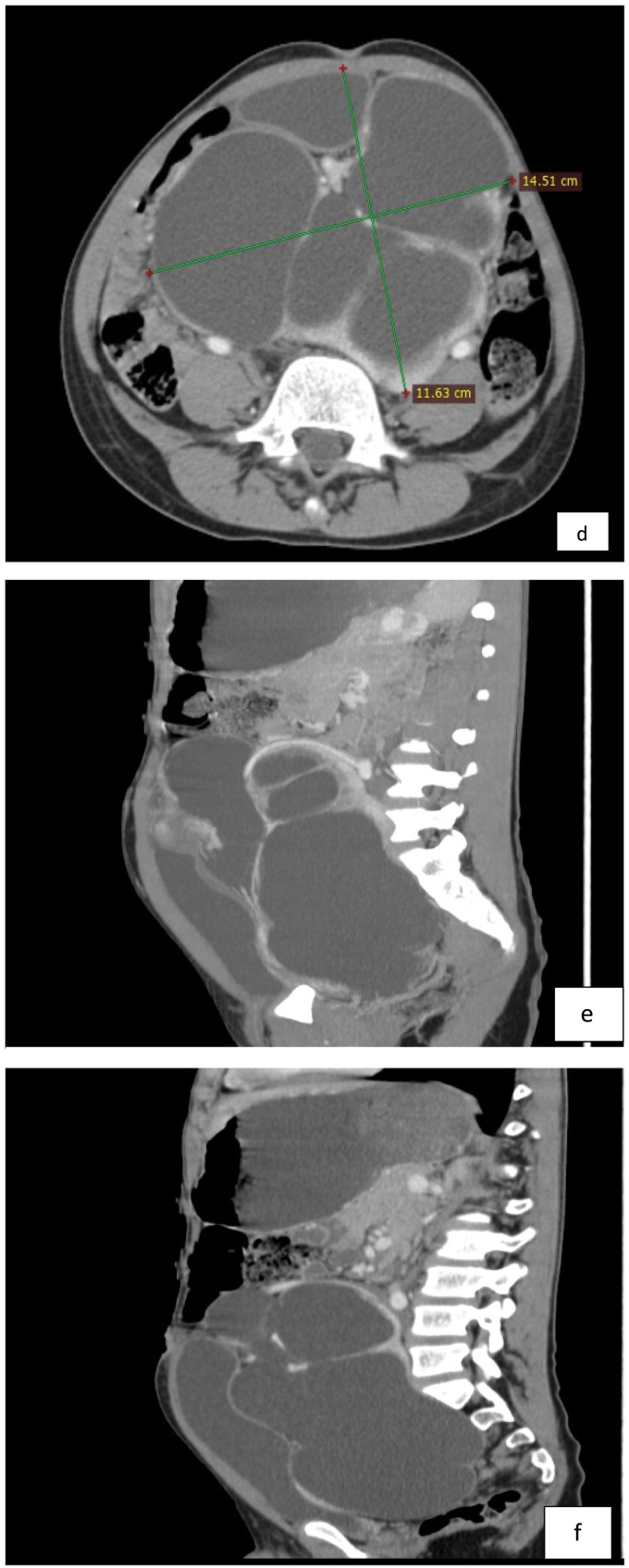

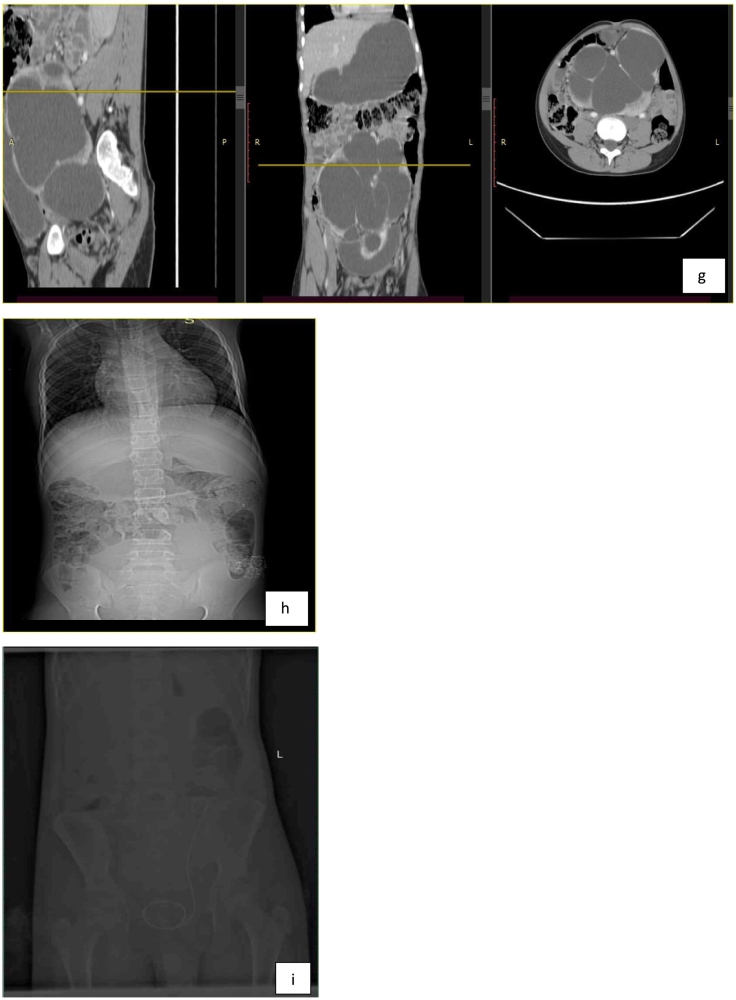
a- the bilateral adrenal glands are elongated and flattened representing ‘pancake’ adrenal gland. b- the bilateral kidneys are not visualized at the renal fossa. c,d- There is a large 14.5 cm*11.6 cm*18.3 cm(AP, ML and CC dimension) central lower abdominal and pelvic region marked dilated communicating pelvicalyceal structure with thinned out enhancing parenchymal cortex and distorted cortical outline. e,f Showed sagittal post contrast arterial phase image shows dilated pelvicalyceal structure with distended urinary bladder (e). Sagittal post contrast arterial phase scan shows arterial supply arising from the distal abdominal aorta (f). g,h sagittal, coronal and axial scan in comparison(g). scout film showed normal lung parenchyma and cardiac shadow(h). i-showed Post pyeloplasty well positioned Double J stent in situ.

Bedside abdominopelvic ultrasound showed a pelvic kidney with severe hydronephrosis, a thinned-out cortex, and echogenic debris. CT-urography showed a severely hydronephrotic pelvic kidney with a thinned-out cortex over most of the kidney, with a thin rim of cortex in the lower pole. The bladder was pushed anteriorly (Fig. 1a–i).

### 1.3 Management

After discussing definitive management options with the family, surgical exploration was decided upon.

Under general anesthesia in the supine position, a transurethral catheter was inserted. The abdomen was prepared and approached via a lower midline vertical incision.

**Fig. 2:**
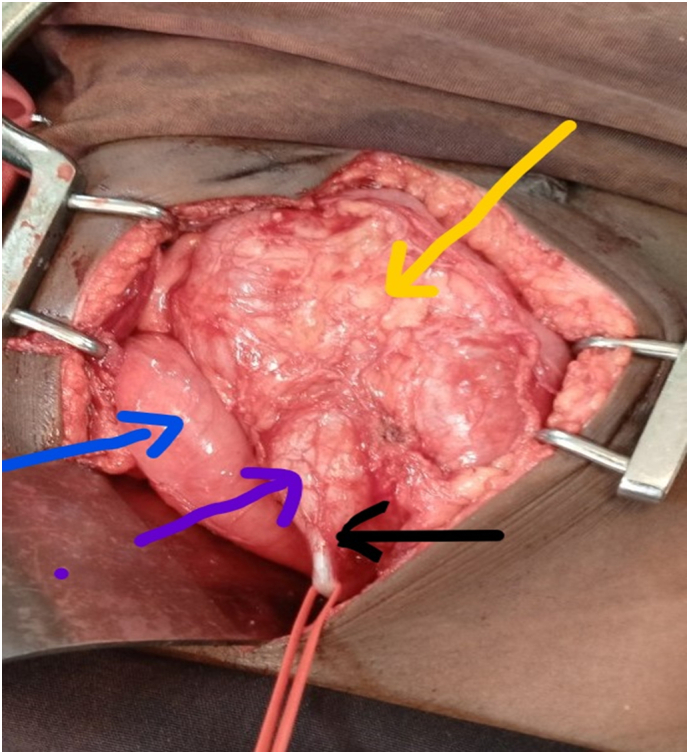
Intraoperative picture showed black arrow-pelviureteric junction looking stenosed. Violate arrow pointed to distended renal pelvis. Blue arrow pointed to distended bladder. Yellow arrow pointed to ballooned renal parenchyma.

**Fig. 3:**
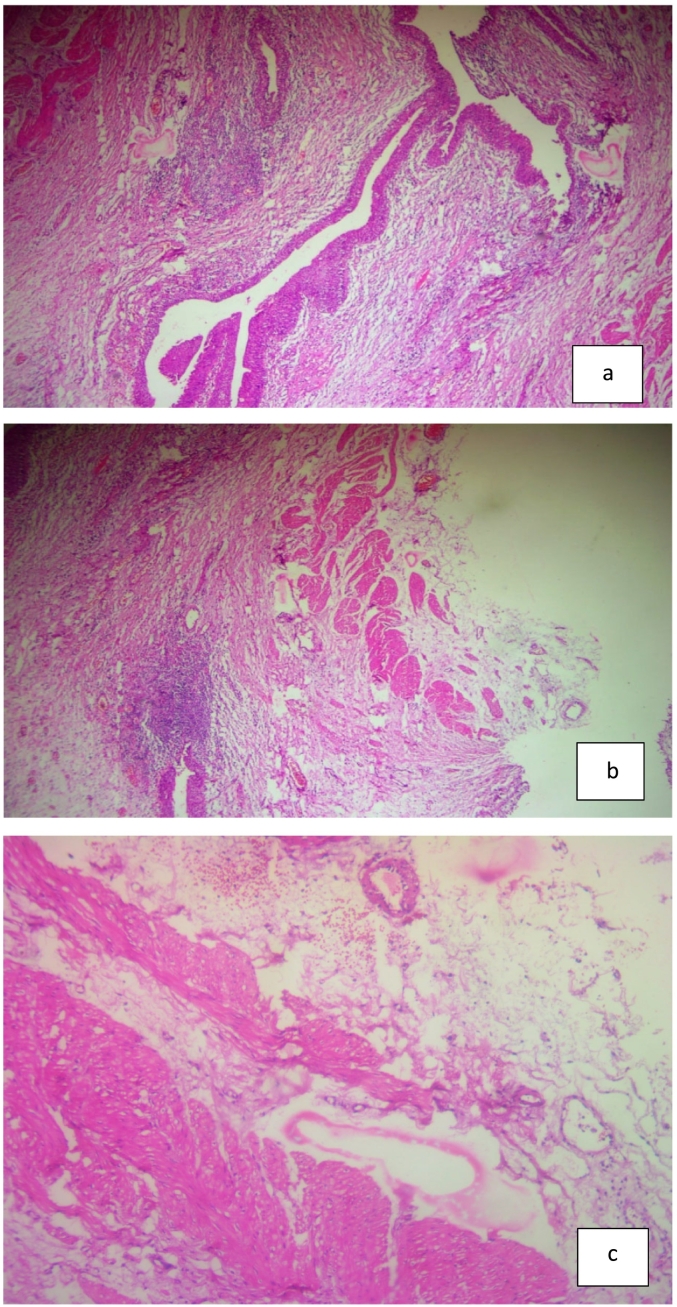
abc showed Pathology-Sections show tissue lined by bland looking urothelial epithelium with underlying edematous lamina propria infiltrated by sheets of lympho-plasma cells and adjacent bundles of hyperplastic smooth muscles. No atypical cells seen.

Findings included a ballooned pelvic kidney, located predominantly on the left side, with a distended pelvis and a proximal ureter with a stent in situ. A stenotic transition was observed between the pelvis and the proximal ureter at the pelvi-ureteral junction (PUJ) (Fig. 2). The ureter was transected at the PUJ (at the stenotic segment) and sent for pathological evaluation (Fig. 3(abc)). The kidney was full of thin pus, which was aspirated and sent for culture. The calyceal system was irrigated with normal saline.

An Anderson-Hynes (AH) pyeloplasty was performed over the stent. An 18Fr nasogastric tube was left as a drain in the pelvic cavity around the anastomotic area.

Intraoperatively, the intra-abdominal cavity was thoroughly examined for another kidney and ureter, but none were found. The bladder was also examined with transurethral flexible cystoscopy to look for a possible second ureteric orifice; however, no orifice was identified.

### 1.4 Follow up

Postoperatively, the patient experienced low-grade fever, chills, and episodic vomiting. Objectively, he was tachycardic and had a low-grade fever; otherwise, his chest was clear, and his abdomen was soft.

Laboratory results were within normal limits, except for creatinine (1.6 mg/dL). Urine and blood cultures were sent. In the meantime, ascending sepsis of genitourinary origin was suspected, and the patient was empirically started on intravenous antibiotics, which were later adjusted based on culture sensitivity results from the intraoperative sample.

A postoperative plain abdominal x-ray (Fig. 1i) was obtained to assess stent position (intraoperative fluoroscopy was unavailable).

Drainage output was nil for two consecutive postoperative days. The transurethral catheter and drain were removed on postoperative days 3 and 4, respectively. Fever, chills, vomiting, and tachycardia resolved, and the patient was discharged on postoperative day 5 with a follow-up clinic appointment.

At two and four weeks postoperatively, the patient was seen in the outpatient clinic and was doing well clinically, with a serum creatinine of 1.5 mg/dL and a well-healing wound. The DJ stent was removed cystoscopically under local anesthesia at the four-week visit.

## 2 Discussion

Solitary pelvic or ectopic kidney is considered a rare anomaly with limited number of reported cases. A literature review by Stevens, done in a 1937 encompassing 27 cases (including two of his own), estimated the incidence to be approximately 1 in 22,000 births [17].

Human renal development begins during the fifth gestational week, involving complex interactions between the ureteric bud (UB), which gives rise to the renal pelvis, ureter, and lower urinary tract, and the metanephric mesenchyme (MM), the origin of the renal parenchyma. Nephron formation continues until the 34th–36th gestational week, ceasing thereafter.

Any insult that affects UB-MM interaction during this critical period can lead to various Congenital Anomalies of the Kidney and Urinary Tract (CAKUT).

Pelvic kidney encompasses a spectrum of congenital anatomical abnormalities resulting from the failure of one or both kidneys to ascend from the pelvis to their normal lumbar position during the metanephric stage of embryogenesis. This abnormal location predisposes patients to a higher risk of traumatic injury, urinary tract infections (UTIs), renal calculi, and other urological complications due to the kidney's exposed position and potentially compromised drainage. However, many individuals with pelvic kidney remain asymptomatic, with the condition often discovered incidentally during imaging performed for unrelated reasons.

The kidney develops between weeks 6 and 8 after conception, and the embryologic kidney rises from the pelvis into the lumbar region in the 9th week. If the kidney fails to pass above the fork of the umbilical arteries, the blood supply degenerates, or there are other factors inhibiting renal migration, then the kidney fails to rise to its normal anatomical location and instead becomes ectopic [2,3].

The exact location can be varied, with most cases being in the contralateral pelvis, but in the cases of crossed renal ectopia, both kidneys can be on the same side of the spine or, more rarely, the kidney can be outside the pelvis or retroperitoneal space entirely and even become located within the thorax [4,5].

The incidence of ectopic kidney quoted is variable worldwide but is often approximately 1 in 1000 births. A retrospective study of 13,701 antenatal scans in Turkey found an incidence of pelvic kidneys of 1 in 571, although this study only included scans with a normal amniotic fluid volume [6].

During normal renal development, the metanephros starts at 5 to 6 weeks after conception. The metanephros would be within the caudal pelvis and migrates from this position to the lumbar region by week 8. If the kidney does not enter the retroperitoneal fossa, it is termed ectopic, and if it remains within the pelvis, it is deemed a pelvic kidney. In very rare instances, the ectopic kidney may be in the thorax. This is usually associated with a diaphragmatic hernia [4].

Most patients with ectopic kidneys are asymptomatic, and if recognized at all, the diagnosis tends to be an incidental finding while investigating other pathology or on routine antenatal ultrasonography. However, urinary tract complications can develop, and patients may present with a range of pathologies, including increased incidence of urinary tract infections, ureteropelvic junction obstruction in the ectopic kidney, or increased risk of renal calculi. The most common associated abnormality is vesicoureteric reflux, which occurs in 30 % of patients with simple renal ectopia [7].

Ureteropelvic junction obstruction (UPJO) is a well-recognized clinical entity that results in impaired urine flow from the renal pelvis into the ureter and, if not detected and treated properly, can result in complete loss of the affected kidney. UPJO is mainly a congenital condition that can be detected by antenatal ultrasound during the second trimester [8,9].

Causes for UPJO can be congenital or acquired. Congenital causes are ureteral hypoplasia may lead to an a peristaltic segment of the ureter, high insertion of the ureter into the renal pelvis, entrapment of the ureter by a crossing accessory renal vesseland rarely malrotated kidney while acquired causes can be due to extrinsic compression or intrinsic lesions. However in many cases, the exact cause of UPJO is not identified. It can affect one or both kidneys and severity of can vary widely, from mild obstruction to complete blockage of urine flow.

Ureteropelvic junction obstruction (UPJO) is more commonly seen in the pediatric age group rather than adults, and this anatomical pathology is seen more frequently in boys than in girls, with up to twice the number of cases in males compared to females. The left side is as well affected twice as often as the right side. It is the most common cause for antenatal detected hydronephrosis at around 80 % of all causes [9].

UPJO is rare entity, has an estimated incidence of 1 in 1000 to 1500 live births. It is more common in pediatrics, that does not make it rare to be seen in adults [10].

Most of the UPJOs seen are partial. In this type of obstruction, there is an increase in the production of the vasoactive peptides and cytokines as interleukin (IL)-5 and eotaxin-2 from the urothelium, acting as a chemoattractant for leukocytes, leading to inflammatory cell infiltration. By changing the eicosanoid elaboration in the kidney, the monocytic infiltration is believed to affect the renal blood supply and decrease the total GFR in the affected kidney. Similarly, the activation of the renin-angiotensin system can cause a reduction in the GFR of the affected kidney by its vasoconstrictor effect [9,10].

Parenchymal damage was observed in severe cases and late presentation of ureteropelvic junction obstruction (UPJO). The lack of clear histological findings is considered one of the major obstacles in the clinical assessment of UPJO [10].

Hitological findings differ based on case of UPJO on study done to Histopathology in Ureteropelvic junction obstruction with and without Crossing Vessels showed increased inflammation in the presence of a crossing vessel but a similar composition of muscle and fibrosis and UPJO with an associated lower-pole vessel may represent a chronic process [10].

Other study found that the pathological changes at UVJ and UPJ segments resemble fetal ureter morphology. They also found that in fetal ureters, as the gestation progressed, there was an increase in the ICC density/smooth muscle, whereas the collagen content decreased. While the entire ureter has uniform embryological origin, it essentially remains an epithelial tube until the late gestation [11].

Diagnosis relies on a comprehensive evaluation, including patient history (e.g., flank pain, UTIs), physical examination, and various imaging modalities. Ultrasound is often the initial imaging study, followed by more detailed investigations such as CT urography, which provides anatomical detail of the collecting system and potential obstructions. Renal scintigraphy (e.g., MAG3 scan) is sometimes used to assess differential renal function and drainage patterns, particularly when management decisions are unclear.

The primary objectives of treatment are to optimize renal drainage, enhance renal function, and alleviate clinical symptoms. When there is indication for intervention, patients can be on close surveillance with serial CT imaging. The long-term management strategy is then tailored based on the patient's clinical course, specifically the development of new or worsening symptoms, and/or evidence of impaired split renal function as demonstrated on diuretic renography.

Surgical intervention, typically pyeloplasty (open, laparoscopic, or robotic-assisted), is indicated for symptomatic patients with significant obstruction and/or declining renal function.

A variety of open and minimally invasive surgical techniques are available for treatment of UPJO. Traditionally open pyeloplasty has been the standard of care but minimally invasive surgical techniques have become increasingly popular. Endopyelotomy has a lower success rate than other modalities (42–90 % depending on the approach), but is associated with reduced pain and shorter convalescence. Laparoscopic pyeloplasty and robot-assisted pyeloplasty have similar success rates to open pyeloplasty (>90 %), with the additional advantages of significantly reduced morbidity and shorter convalescence [12].

In our case, patient presentation was late and most of kidney parenchyma was already gone, only rim of tissue left, and serum creatinine was trending up and CT urography was not typical of UPJO(Dilated renal pelvis is described as “ballooned renal pelvis” with collapsed proximal ureter) [16].

Given the rarity of UPJO in the setting of a solitary pelvic kidney, this diagnosis should be considered in patients presenting with obstructive uropathy. Differentials could be pancake kidney, fused crossed ectopia with component atrophy.

## 3 Weaknesses and limitations

Renal scintigraphy was not performed in our patient due to the unavailability of the modality. While CT urography revealed no gross anatomical abnormalities suggestive of ectopic renal tissue, renal scintigraphy would have provided further assurance in excluding its presence. This might be taken as limitation of our case report.

Due to the lack of expertise and resources for minimally invasive techniques, our patient was managed with open Anderson-Hynes pyeloplasty.

## 4 Conclusion

Congenital solitary pelvic kidney complicated by UPJO represents a rare and challenging clinical scenario that carries a substantial risk of progressive renal parenchymal loss and eventual decline in overall renal function, emphasizing the need for careful evaluation and tailored management strategies.

Imaging might not be straight forward in pelvic kidney with UPJO specially patients who present late. Thus, UPJO should considered in patients with pelvic kidney as cause of loss of renal function. Early diagnosis and management are crucial for preserving renal parenchyma.

## CRediT authorship contribution statement

1. Abiy Tadele Alene (MD, Final year Urology Resident): Assisted surgery, diagnosed the patient, Conceptualization, Methodology, Manuscript writing, and Submission and followed the patient.
2. Habtamu Aderaw Zeru (MD, Assistant Professor of urology): Leading Surgeon and reviewed the final manuscript.
3. Tensae Tesfaye Amare (MD, final year pathology resident): Diagnosed histology and reviewed the final manuscript.
4. Biniam Goa Mammo,(MD, Final year clinical Radiology resident); Reviewed manuscript, involved in CT scan image reading.
5. Yohannes Kifle Tesema,(MD, Urology resident): Assisted surgery Involved in patient diagnosis and follow up.
6. Biniam Yohannes Kassa (MD Final year internal medicine Resident, Nephrology Unit); Reviewed manuscript involved in follow up and management of patient.

## Ethical approval

Ethical approval was provided by the author's institution.

Ethical review committee of the Department of Surgery, College of Health Sciences, School of Medicine, Addis Ababa University in March 2025 G.c with reference No. Dos/Rec/120/2025/2017

## Guarantor

1. Abiy Tadele Alene (MD, Final year Urology Resident)

## Funding

There is no source of funding found for this research paper.

## Patient (parent's) consent

Written informed consent was obtained from the patient parent (mother) for publication of this case report and accompanying images.

A copy of the written consent is available for review by the Editor-in-Chief of this journal on request.

## Declaration of competing interest

All authors declare that they have no conflict of interest.
